# Depression Screening Positivity, Treatment-Seeking Intention, and Willingness to Take Antidepressants in a Self-Selected Online Sample of Korean Adults: Cross-Sectional Questionnaire Study

**DOI:** 10.2196/96178

**Published:** 2026-11-11

**Authors:** Ha-Na Kim, Chul-Min Kim, Young Kyu Park, Kiheon Lee, Youn Huh, Wonsuk Kim, Young Sik Kim

**Affiliations:** 1 Department of Family Medicine, College of Medicine, The Catholic University of Korea St. Vincent's Hospital, Seoul, Republic of Korea; 2 Department of Family Medicine, College of Medicine, The Catholic University of Korea Seoul St. Mary's Hospital, Seoul, Republic of Korea; 3 Occupation and Environment Medicine, Yeosu Cheonnam Hospital, Yeosu, Republic of Korea; 4 Department of Family Medicine, School of Medicine, Seoul National University Bundang Hospital, Seongnam, Gyeonggi-do, Republic of Korea; 5 SafeAI, Seoul, Republic of Korea; 6 School of Electrical Engineering, Korea University, Seoul, Seoul, Republic of Korea; 7 Department of Family Medicine, College of Medicine, University of Ulsan, 88, Olympic-ro 43-gil, Songpa-gu, Seoul, Ulsan, 05505, Republic of Korea, 82 2-423-3830

**Keywords:** depression, depression screening, help-seeking, treatment-seeking intention, antidepressants, barriers, South Korea

## Abstract

**Background:**

Depression is a prevalent psychiatric condition with functional and socioeconomic consequences. Understanding the barriers to professional help-seeking and treatment acceptance among individuals with depressive symptoms is important.

**Objective:**

This study aimed to quantify depression screening positivity and examine treatment-seeking intention, willingness to use antidepressants, and perceived barriers among Korean adults, with attention to prior depression-related health care experiences.

**Methods:**

A cross-sectional online survey was conducted in South Korea (March 16-April 5, 2024) via paid social media advertising and messaging app invitations. A total of 1053 adults aged 20 years and older participated in this study. Depression screening was conducted using 2 Primary Care Evaluation of Mental Disorders–derived items, with the endorsement of either item defining a positive screen. History of depression diagnosis or treatment, intentions toward professional treatment, antidepressant use, and perceived barriers were assessed.

**Results:**

Overall, 65% (684/1053) of the participants had a positive depression screen. A positive depression screen was not associated with the intention to seek professional treatment for depression (adjusted odds ratio [aOR] 1.04, 95% CI 0.77‐1.41). Participants with a positive depression screen had lower odds of being willing to take antidepressants than those with a negative screen (aOR 0.73, 95% CI 0.54‐0.99). A within-participant analysis showed that the association of depression screening status differed between treatment-seeking intention and willingness to take antidepressants (ratio of odds ratios 0.70, 95% CI 0.53‐0.94; interaction *P*=.02). The joint response of intending to seek treatment while being unwilling to take antidepressants was more common among participants with a positive than a negative depression screen (75/384, 11% vs 23/369, 6.2%; aOR 1.70, 95% CI 1.04‐2.77). A history of depression diagnosis or treatment was positively associated with treatment-seeking intention and willingness to use antidepressants. Among screen-positive adults, those without such a history more often reported that they would not seek treatment if diagnosed with depression (129/505, 25.5% vs 26/179, 14.5%; *P*=.002) or would be unwilling to take antidepressants if recommended (154/505, 30.5% vs 29/179, 16.2%; *P*<.001). Barrier profiles to treatment seeking differed by history of depression diagnosis or treatment (interaction *P*<.001), whereas the patterns of reasons for unwillingness to take antidepressants did not differ according to history (interaction *P*=.08).

**Conclusions:**

In this online survey sample, a positive depression screen was associated with a lower willingness to use antidepressants but not with treatment-seeking intention. Among screen-positive participants, those without prior depression-related health care experience had lower help-seeking intention and willingness to take antidepressants than those with prior contact, suggesting the need to reduce barriers to care and support earlier treatment initiation.

## Introduction

Depression is a disorder characterized by persistent feelings of sadness, loss of interest or pleasure, reduced energy, and accompanying cognitive and physical symptoms that noticeably impair daily functioning [1]. It not only diminishes the quality of life but also imposes a social burden through reduced academic and occupational functioning and an increased risk of comorbidities [2,3]. Worldwide, approximately 5.7% of the adult population is estimated to have depressive disorders [4]. In South Korea, the lifetime prevalence of depressive disorders among adults has been steadily increasing [5], and cross-national surveys have reported a high prevalence of depressive disorders in the country [6,7], raising socioeconomic concerns [8].

Depression can become chronic and has a high rate of recurrence [9]; therefore, early identification followed by appropriate clinical assessment and intervention is important for improving treatment outcomes [10,11]. Brief symptom-based screening provides a rapid and low-burden method to identify individuals who may benefit from further evaluation. Such screening complements formal clinical assessment based on standardized diagnostic criteria [12,13], and its clinical value depends on whether individuals who screen positive proceed to professional assessment and treatment [14]. Appropriate medication and professional intervention in the early stages of the disease help preserve neural plasticity in the brain [15,16], prevent symptom worsening [11], and accelerate the patient’s return to social functioning [17]. It has been reported that the longer the delay between symptom onset and the start of treatment, the greater the severity of symptoms, the poorer the treatment response, and the higher the risk of chronicity and suicidal behavior [18], leading to a vicious cycle of worsening physical health and social isolation [19,20]. Despite the importance of early intervention, treatment is often delayed by personal and structural barriers [21,22], and a significant gap exists between diagnosis and treatment in clinical settings [23]. In previous studies, the annual treatment rate for major depressive episodes was reported to be only 39.2% [24], and the treatment gap was reported to be 56.3% [25], with the proportion of patients receiving antidepressants ranging from 0% to 38% across primary care settings [26].

Since the onset of the COVID-19 pandemic, an increase in depressive symptoms and psychological distress has been reported in South Korea. This widespread deterioration in mood, colloquially termed “corona blue” [27], has further increased the importance of identifying individuals who may benefit from clinical assessments. From this perspective, it is important to understand whether individuals with a positive depression screen intend to seek professional care, are willing to take antidepressants, and encounter barriers to engaging with treatment; however, evidence on these issues remains limited in South Korea [28]. Therefore, using a depression screening tool, this study aimed to quantify depression screening positivity; examine treatment-seeking intention, willingness to take antidepressants, and perceived barriers; and compare these outcomes according to prior depression-related health care experience in a self-selected online convenience sample of Korean adults recruited through social media advertising and messaging app invitations.

## Methods

### Study Design

This cross-sectional study used a self-administered, web-based questionnaire hosted on Walla (Paprika Data Lab Inc; walla.my [29]), a Korean online survey platform (Table S1 in Multimedia Appendix 1). The questionnaire comprised 7 core items and 2 conditional multiple-response items; mobile phone numbers were also collected. Items were presented in a fixed order over multiple screens; conditional questions were shown through the platform’s skip logic only to respondents for whom they were applicable, so the number of screens varied between respondents. All applicable questions were required for submission, and respondents could review and revise their answers before submission. The electronic questionnaire was not pilot-tested before deployment.

### Participant Recruitment

Participants were recruited from March 16 to April 5, 2024, through a paid Instagram advertisement and survey invitations shared via KakaoTalk (Kakao Corp), resulting in a self-selected nonprobability online sample. The advertisement targeted users in Seoul aged 18 years and older with health-related interests. The advertisement described the survey purpose, the expected completion time of approximately 1 minute, and the opportunity to enter a prize draw. The advertisement reached 6856 accounts, generated 11,662 impressions, and received 685 link clicks. These metrics covered only the paid advertisement; additional access through KakaoTalk, the account’s profile link, and direct URL entry prevented determination of the total number of unique individuals exposed to the invitation across all recruitment channels. Therefore, a response rate could not be calculated. The platform’s duplicate response restriction was enabled. We additionally checked mobile phone numbers and excluded 28 duplicate submissions, retaining only the first submission for each number. Of the remaining 1064 questionnaires, 11 from respondents aged below 20 years were excluded, leaving 1053 complete questionnaires for analysis.

### Ethical Considerations

This study was approved by the Institutional Review Board of the Catholic University of Korea (approval VC24ZISI0127). The institutional review board waived the requirement for written informed consent. Mobile phone numbers were used for duplicate submission checks and voucher delivery and were removed from the dataset before statistical analysis. Thirty respondents received a coffee voucher worth approximately US $3 each through a prize draw.

### Measurements

Information on age, sex (male or female), and history of depression diagnosis or treatment was obtained through the questionnaire, along with a 2-question instrument for screening for depression derived from the Primary Care Evaluation of Mental Disorders (PRIME-MD): (1) “During the past month, have you often been bothered by feeling down, depressed, or hopeless?” and (2) “During the past month, have you often been bothered by little interest or pleasure in doing things?” [30,31]. The 2-item depression screening questions from the PRIME-MD have demonstrated high sensitivity but modest specificity for detecting depression, supporting its use as a brief screening tool [31,32]. Participants who endorsed either item were classified as having a positive depression screen [31].

Treatment-seeking intention and willingness to take antidepressants were assessed using 2 hypothetical yes-or-no questions. Participants were asked whether they would seek treatment at a hospital if they were diagnosed with depression and whether they would take antidepressants if a physician recommended them for the treatment of depression. Participants who responded “no” to either question were asked to select all applicable reasons for not seeking treatment or for being unwilling to take antidepressants, respectively, using a multiple-response format. The response options for perceived barriers to seeking treatment for depression were as follows: “I cannot accept a diagnosis of depression,” “I believe I can handle it on my own,” “I am concerned about negative views or stigma from others,” “I am worried that it could interfere with my school or work life,” “I am concerned that having a medical record could cause problems later (e.g., when applying for insurance),” “I do not think that treatment at a hospital would be effective,” “I do not have a trusted hospital/clinic or regular physician,” “I am concerned about medication side effects,” and “I lack the time or financial resources for treatment.” The response options for the reasons for a negative response to taking antidepressants were as follows: “I am concerned about medication side effects,” “I am concerned about negative views or stigma from others regarding antidepressant use,” “I prefer non-pharmacological treatments (e.g., psychotherapy) over antidepressants,” “I feel reluctant or anxious about taking antidepressants,” “I am concerned that having a medical record could cause problems later (e.g., when applying for insurance),” and “I am concerned that antidepressants might be addictive or lead to dependence.

### Statistical Analysis

We used the chi-square test to analyze the differences in depression screening positivity and attitudes toward help-seeking and depression treatment. Multivariable logistic regression analyses were used to assess the associations between both depression screening status and history of depression diagnosis or treatment and treatment-seeking intention and willingness to take antidepressants, adjusting for age and sex as covariates. To compare the associations between both depression screening status and treatment-seeking intention and willingness to take antidepressants within participants, the 2 binary outcomes were stacked and analyzed using repeated-outcome generalized estimating equations with participant-level clustering, an exchangeable working correlation structure, and robust SEs. The model included outcome type, depression screening status, their interaction, age, sex, and the interactions of outcome type with age and sex. As exploratory analyses, we compared the joint response pattern of intending to seek treatment but being unwilling to take antidepressants, willingness to take antidepressants among participants intending to seek treatment, and willingness to take antidepressants among screen-positive participants intending to seek treatment according to the history of depression diagnosis or treatment, using logistic regression adjusted for age and sex. Barriers to seeking treatment for depression and taking antidepressants were assessed using multiple-response items; endorsement of each item was coded as a binary variable and restructured into a participant-item long format. Age- and sex-adjusted logistic generalized estimating equation models with participant-level clustering, exchangeable correlation, and robust SEs tested the history of depression diagnosis or treatment-by-item interaction using a joint Wald test. Item-specific age- and sex-adjusted logistic regressions were followed by separate Benjamini-Hochberg corrections within each barrier domain to control the false discovery rate at 5%. Statistical analyses were performed using SPSS Statistics (version 18.0; IBM Corp) for Windows and Python (version 3.13.5; Python Software Foundation) with *statsmodels* (version 0.14.6). A *P* value below .05 was considered statistically significant.

## Results

### Demographic Characteristics and Response of Participants According to Depressive Screening Status

As shown in Table 1, a total of 65% (684/1053) of the participants met the study definition of a positive depression screen (229/408, 56.1% for male individuals and 455/645, 70.5% for female individuals). Screening positivity was more frequent among participants with a history of diagnosed depression or treatment than among those without (*P*<.001). Among all participants, 77.1% (812/1053) said that they would receive treatment at a hospital if they were diagnosed with depression, and 75.6% (796/1053) said that they would take antidepressants if they had to treat depression. Among participants with a positive depression screen, unwillingness to take antidepressants was more prevalent than among those with a negative screen (*P*=.02). The proportion of participants who screened positive did not differ significantly by age (*P*=.08) but differed significantly by sex (*P*<.001).

**Table 1.** Characteristics and survey responses by depression screening status among adults in a cross-sectional online survey, South Korea, March-April 2024 (N=1053).

Variables	Total, n (%)	Depression screening status, n (%)		
		Positive (n=684)	Negative (n=369)	*P* value
Age (y)				.08
20-29	243 (23.1)	146 (60.1)	97 (39.9)	
30-39	397 (37.7)	272 (68.5)	125 (31.5)	
40-49	267 (25.4)	179 (67.0)	88 (33.0)	
50-59	104 (9.9)	65 (62.5)	39 (37.5)	
≥60	42 (4.0)	22 (52.4)	20 (47.6)	
Sex				<.001
Female	645 (61.3)	455 (70.5)	190 (29.5)	
Male	408 (38.7)	229 (56.1)	179 (43.9)	
History of depression diagnosis or treatment				<.001
Yes	218 (20.7)	179 (82.1)	39 (17.9)	
No	835 (79.3)	505 (60.5)	330 (39.5)	
Intention to seek professional treatment if diagnosed with depression				.82
Yes	812 (77.1)	529 (65.1)	283 (34.9)	
No	241 (22.9)	155 (64.3)	86 (35.7)	
Willingness to take antidepressants if prescribed				.02
Yes	796 (75.6)	501 (62.9)	295 (37.1)	
No	257 (24.4)	183 (71.2)	74 (28.8)	

### Associations Between Both Depression Screening Status and History of Depression Diagnosis or Treatment and Treatment-Seeking Intention and Willingness to Take Antidepressants

In age- and sex-adjusted analyses, a positive depression screen was not significantly associated with treatment-seeking intention (adjusted odds ratio [aOR] 1.04, 95% CI 0.77‐1.41; *P*=.80). Participants with a positive screen had lower odds of willingness to take antidepressants (aOR 0.73, 95% CI 0.54‐0.99; *P*=.047). A history of depression diagnosis or treatment was positively associated with both treatment-seeking intention for depression and willingness to take antidepressants (Table 2).

**Table 2.** Associations between both depression screening status and history of depression diagnosis or treatment and treatment-seeking intention and willingness to take antidepressants among 1053 adults in a cross-sectional online survey, South Korea, March to April 2024. Values were adjusted for age and sex.

	Treatment-seeking intention for depression				Willingness to take antidepressants				
	Unadjusted OR^a^ (95% CI)	*P* value	Adjusted OR (95% CI)	*P* value	Unadjusted OR (95% CI)		*P* value	Adjusted OR (95% CI)	*P* value
Depression screening status									
Negative	1	—^b^	1	—	1		—	1	—
Positive	1.04 (0.77-1.40)	.81	1.04 (0.77-1.41)	.80	0.69 (0.51-0.93)		.02	0.73 (0.54-0.99)	.047
History of depression diagnosis or treatment									
No	1	—	1	—	1		—	1	—
Yes	2.12 (1.39-3.21)	<.001	2.18 (1.43-3.30)	<.001	1.97 (1.33-2.93)		.001	2.14 (1.44-3.20)	<.001

a OR: odds ratio.

b *P* values are not applicable to the reference categories.

A within-participant comparison showed a significant interaction between depression screening status and the type of outcome—treatment-seeking intention vs willingness to take antidepressants (ratio of odds ratios 0.70, 95% CI 0.53‐0.94; interaction *P*=.02), indicating that the association with depression screening status differed significantly between treatment-seeking intention and willingness to take antidepressants (Table S2 in Multimedia Appendix 1). The joint response of intending to seek professional treatment but being unwilling to take antidepressants was more common among participants with a positive screen than among those with a negative screen (75/684, 11% vs 23/369, 6.2%; aOR 1.70, 95% CI 1.04‐2.77; *P*=.04). Among participants who intended to seek treatment, those with a positive screen had lower odds of willingness to take antidepressants than those with a negative screen (aOR 0.59, 95% CI 0.36‐0.97; *P*=.04; Table 3 and Table S3 in Multimedia Appendix 1). Among screen-positive participants who intended to seek professional treatment, a history of depression diagnosis or treatment was associated with a greater willingness to take antidepressants (aOR 2.54, 95% CI 1.32‐4.89; *P*=.005; Tables 3 and 4 and Table S4 in Multimedia Appendix 1).

**Table 3.** Exploratory analyses of treatment-seeking intention and willingness to take antidepressants according to depression screening status and history of depression diagnosis or treatment among adults in a cross-sectional online survey, South Korea, March-April 2024^a^.

Analysis	Participants, n/N (%)	Unadjusted OR^b^ (95% CI)	*P* value	Adjusted OR (95% CI)^c^	*P* value
Intention to seek treatment but unwillingness to take antidepressants: positive vs negative depression screen	75/684 (11.0) vs 23/369 (6.2)	1.85 (1.14‐3.01)	.01	1.70 (1.04‐2.77)	.04
Willingness to take antidepressants among participants intending to seek treatment: positive vs negative depression screen	454/529 (85.8) vs 260/283 (91.9)	0.54 (0.33‐0.88)	.01	0.59 (0.36‐0.97)	.04
Willingness to take antidepressants among screen-positive participants intending to seek treatment: history of depression diagnosis or treatment (yes vs no)	141/153 (92.2) vs 313/376 (83.2)	2.37 (1.24‐4.52)	.009	2.54 (1.32‐4.89)	.005

a Odds ratios compare the first group listed with the group listed after “vs.”

b OR: odds ratio.

c Adjusted for age and sex.

**Table 4.** Associations between history of depression diagnosis or treatment and endorsement of barriers to treatment seeking among adults with a positive depression screen in a cross-sectional online survey, South Korea, March-April 2024^a^.

Items	aOR^b^ (95% CI)^c^	*P* value^d^	Adjusted *P* value^e^
I cannot accept a diagnosis of depression.	0.86 (0.27‐2.69)	.79	.85
I believe I can handle it on my own.	0.37 (0.19‐0.72)	.003	.03
I am concerned about negative views or stigma from others.	0.38 (0.13‐1.11)	.08	.35
I am worried it may interfere with my school or work life.	0.46 (0.18‐1.21)	.12	.35
I am concerned that having a medical record may cause problems later (eg, when applying for insurance).	0.93 (0.43‐2.02)	.85	.85
I do not think treatment at a hospital would be effective.	1.08 (0.54‐2.15)	.83	.85
I do not have a trusted hospital/clinic or regular physician.	1.29 (0.52‐3.23)	.58	.85
I am concerned about medication side effects.	1.51 (0.68‐3.33)	.30	.68
I lack the time or financial resources for treatment.	0.81 (0.36‐1.83)	.62	.85

a Participants without a history of depression diagnosis or treatment served as the reference group.

b aOR: adjusted odds ratio.

c Adjusted for age and sex.

d *P* values from the age- and sex-adjusted models.

e Benjamini-Hochberg–adjusted *P* values for multiple comparisons.

### Treatment-Seeking Intention and Willingness to Take Antidepressants by History of Depression Diagnosis or Treatment Among Adults With a Positive Depression Screen

Among participants with a positive depression screen, 14.5% (26/179) of respondents with a history of depression diagnosis or treatment and 25.5% (129/505) of those without such a history responded that they would not seek treatment if they were diagnosed with depression (*P*=.002). When asked whether they would take antidepressants if recommended, 16.2% (29/179) of screen-positive participants with a history of depression diagnosis or treatment and 30.5% (154/505) of those without such a history responded that they would be unwilling to take antidepressants (*P*<.001; Figure 1).

**Figure 1.**
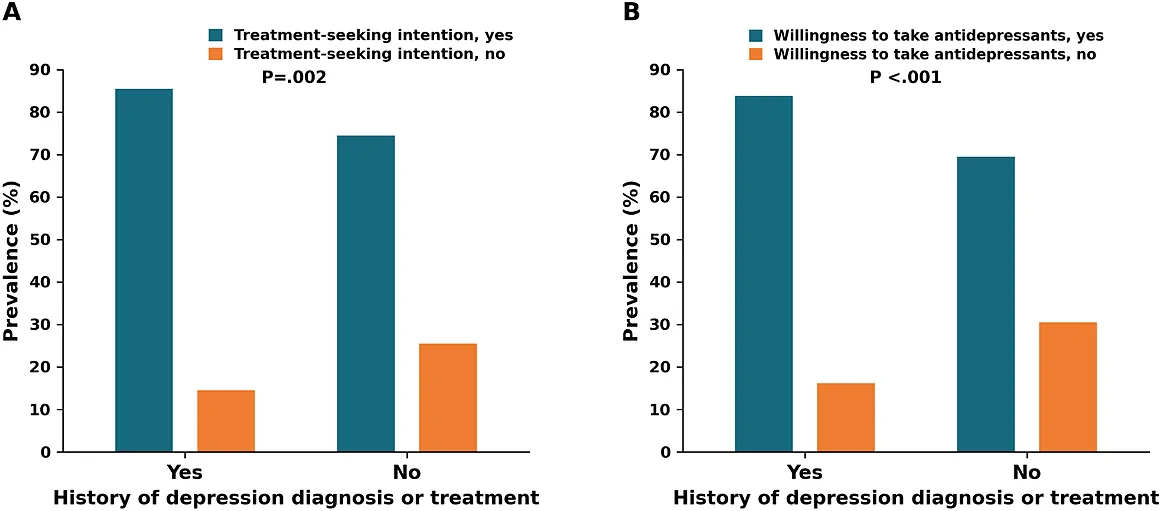
Treatment-seeking intention and willingness to take antidepressants by history of depression diagnosis or treatment among 684 adults with a positive depression screen in a cross-sectional online survey, South Korea, March to April 2024: (A) intention to seek treatment if diagnosed with depression and (B) willingness to take antidepressants if recommended by a physician. Values are percentages within each history group.

### Reasons for Not Seeking Treatment for Depression and Unwillingness to Take Antidepressants According to History of Depression Diagnosis or Treatment Among Adults With a Positive Depression Screen

Among adults with a positive depression screen, the patterns of reasons for not seeking treatment for depression differed significantly between participants with and without a history of depression diagnosis or treatment (group-by-item interaction *P*<.001; Figure 2). At the item level, participants with a history of depression diagnosis or treatment had lower odds of endorsing the statement “I believe I can handle it on my own” than those without such a history (aOR 0.37, 95% CI 0.19‐0.72; adjusted *P*=.03; Table 4).

**Figure 2.**
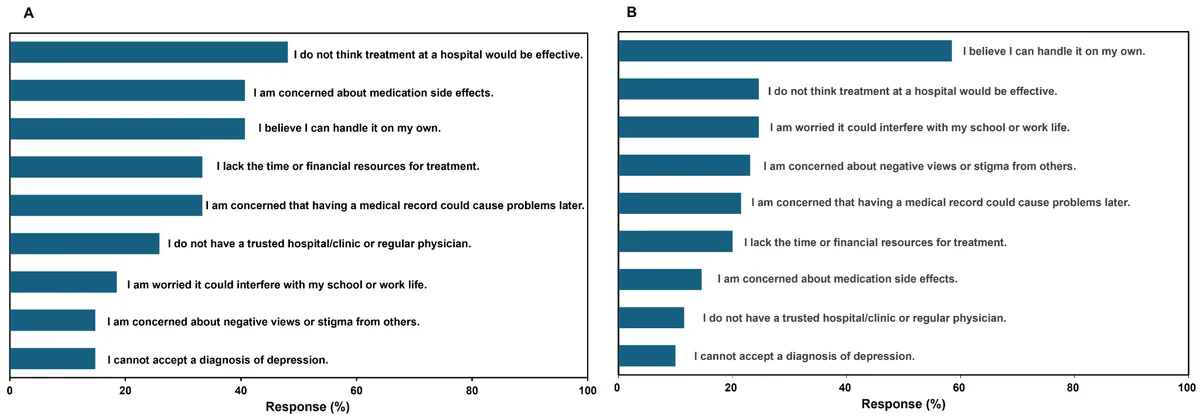
Reasons for not intending to seek treatment if diagnosed with depression by history of depression diagnosis or treatment among adults with a positive depression screen in a cross-sectional online survey, South Korea, March to April 2024: (A) history of depression diagnosis or treatment and (B) no history of depression diagnosis or treatment. The analysis included 155 screen-positive participants who reported no intention to seek treatment. Values are percentages within each history group; multiple responses were permitted. The group-by-item interaction was evaluated using a generalized estimating equation logistic regression model (*P*<.001).

The patterns of reasons for being unwilling to take antidepressants did not differ by history of depression diagnosis or treatment (group-by-item interaction *P*=.08; Figure 3). At the item level, participants with a history of depression diagnosis or treatment had lower odds of endorsing the statements “I am concerned about medication side effects,” “I am concerned about negative views or stigma from others regarding antidepressant use,” “I prefer non-pharmacological treatments (e.g., psychotherapy) over antidepressants,” and “I am concerned that antidepressants might be addictive or lead to dependence” than those without such a history (aOR 0.43, 95% CI 0.24‐0.76; aOR 0.35, 95% CI 0.16‐0.75; aOR 0.45, 95% CI 0.22‐0.91; and aOR 0.30, 95% CI 0.13‐0.72, respectively; Table 5).

**Figure 3.**
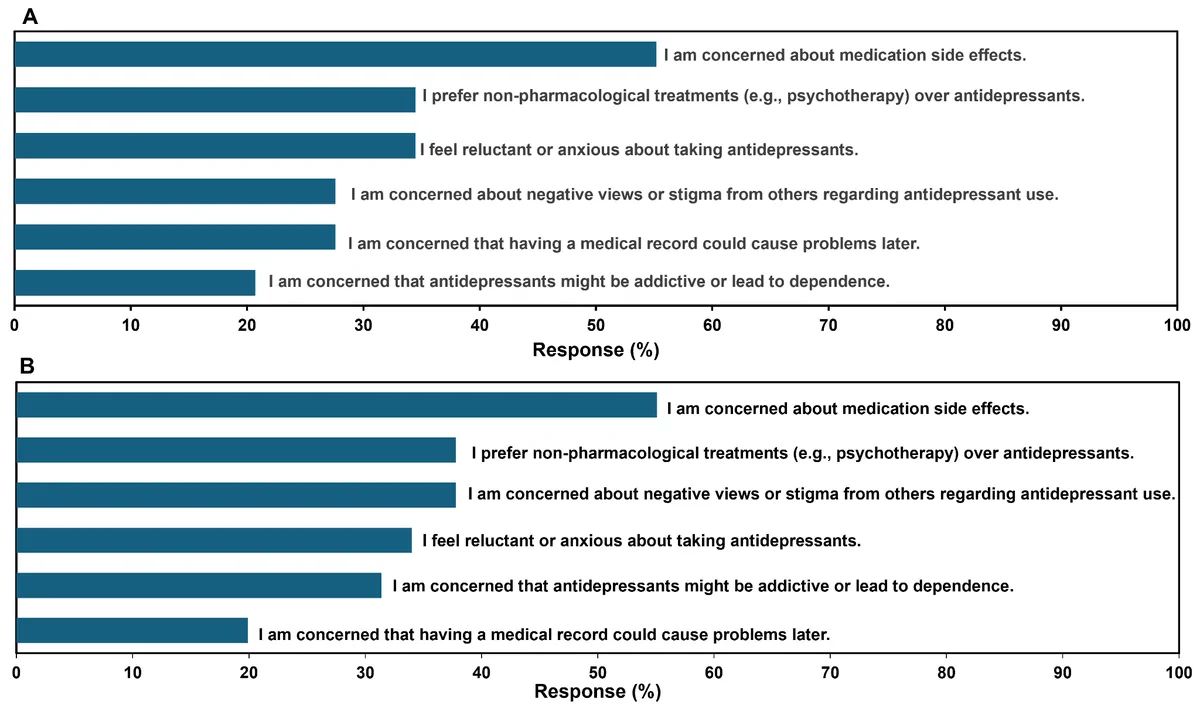
Reasons for unwillingness to take antidepressants if recommended by a physician by history of depression diagnosis or treatment among adults with a positive depression screen in a cross-sectional online survey, South Korea, March to April 2024: (A) history of depression diagnosis or treatment and (B) no history of depression diagnosis or treatment. The analysis included 183 screen-positive participants who reported unwillingness to take antidepressants. Values are percentages within each history group; multiple responses were permitted. The group-by-item interaction was evaluated using a generalized estimating equation logistic regression model (*P*=.08).

**Table 5.** Associations between history of depression diagnosis or treatment and endorsement of barriers to antidepressant use among adults with a positive depression screen in a cross-sectional online survey, South Korea, March-April 2024^a^.

Items	aOR^b^ (95% CI)^c^	*P* value^d^	Adjusted *P* value^e^
I am concerned about medication side effects.	0.43 (0.24‐0.76)	.004	.01
I am concerned about negative views or stigma from others regarding antidepressant use.	0.35 (0.16‐0.75)	.007	.02
I prefer non-pharmacological treatments (e.g., psychotherapy) over antidepressants.	0.45 (0.22‐0.91)	.03	.04
I feel reluctant or anxious about taking antidepressants.	0.51 (0.25‐1.02)	.06	.07
I am concerned that having a medical record may cause problems later (e.g., when applying for insurance).	0.77 (0.34‐1.71)	.52	.52
I am concerned that antidepressants may be addictive or lead to dependence and that I may not be able to stop taking them.	0.30 (0.13‐0.72)	.007	.01

a Participants without a history of depression diagnosis or treatment served as the reference group.

b aOR: adjusted odds ratio.

c Adjusted for age and sex.

d *P* values from the age- and sex-adjusted models.

e Benjamini-Hochberg–adjusted *P* values for multiple comparisons.

## Discussion

### Principal Findings

This study investigated depression screening positivity and attitudes toward seeking treatment for depression in a self-selected online sample of 1053 Korean adults. A total of 65% (n=684) of the participants had a positive depression screen. In age- and sex-adjusted analyses, depression screening positivity was not significantly associated with treatment-seeking intention and showed an association with a lower willingness to take antidepressants. In the within-participant analysis, depression screening status showed different patterns of association with treatment-seeking intention and willingness to take antidepressants, and the joint pattern of intending to seek treatment but being unwilling to take antidepressants was more common among participants with a positive screen. A history of depression diagnosis or treatment was associated with greater treatment-seeking intention and willingness to take antidepressants; among screen-positive participants who intended to seek treatment, such a history was also associated with greater willingness to take antidepressants. Among participants with a positive depression screen, the patterns of reasons for not seeking depression treatment differed significantly between participants with and without a history of depression diagnosis or treatment, whereas the patterns of reasons for unwillingness to take antidepressants did not differ significantly according to a history of depression diagnosis or treatment.

### Interpretation of Findings

Previous studies and Organisation for Economic Co-operation and Development reports have indicated a substantial burden of depressive symptoms in South Korea [6,7]. Potential contributors include employment insecurity, low income, and limited social support, which have been associated with depressive symptoms in the Korean population [33,34]. At the same time, antidepressant consumption in South Korea is among the lowest in Organisation for Economic Co-operation and Development countries [35,36]. In this study, because depression screening positivity was assessed using a screening instrument, the estimated rate might have been higher than that of clinically diagnosed depression [37]. Nonetheless, the results of this study indicate that the rate of positive depression screens and the marked treatment gap might underscore low service use for depressive symptoms in South Korea. Key barriers include stigma and fear of discrimination (public prejudice and structural discrimination), as well as concerns about adverse drug effects and cost and limited accessibility, which were previously identified as prominent obstacles to psychiatric help-seeking in South Korea [22]. In addition, low mental health literacy and shame-related social norms might further discourage disclosure and formal help-seeking, and depressive distress can present with prominent somatic symptoms, potentially delaying recognition and treatment initiation [28,38].

Treatment-seeking intention for depression was not associated with depression screening status, whereas a history of depression diagnosis or treatment was associated with greater intentions to seek treatment for depression, suggesting that there may be a disconnect between the current symptom burden and treatment-seeking intention, whereas prior contact with mental health care services appears to be accompanied by greater willingness to seek treatment, and indicating that individuals without prior contact with care might face barriers to entering the treatment pathway. In addition, participants with a history of depression diagnosis or treatment were less likely to endorse the statement “I believe I can handle it on my own” than those without such a history, suggesting that those with a prior depression-related health care experience could have a greater recognition of depression as a condition that warrants professional care and possibly reflecting higher mental health literacy [39,40].

Willingness to take antidepressants was negatively associated with a positive depression screen; however, a history of depression diagnosis or treatment was associated with a greater willingness to take antidepressants, suggesting that attitudinal barriers to antidepressant use persist among individuals with a positive depression screen and prior contact with depression-related health care services appears to be accompanied by greater acceptance of pharmacotherapy. The overall reason profiles for unwillingness to take antidepressants did not differ according to a history of depression diagnosis or treatment; however, at the item level, prior depression-related health care experience was associated with less endorsement of negative statements about antidepressants, indicating greater acceptability [41].

Among participants with a positive depression screen, willingness to seek professional care did not always extend to willingness to take antidepressants; a subset reported that they would seek treatment but would not take antidepressants if recommended. This pattern suggests that medication acceptance may represent an additional barrier beyond initial willingness to seek care [42], although the survey assessed hypothetical intentions rather than actual care entry or treatment behavior. The greater willingness to take antidepressants among participants with a history of depression diagnosis or treatment may reflect greater familiarity with depression care or preexisting receptivity. Therefore, medication-specific concerns, including fears of adverse effects and dependence, may warrant discussion during treatment consultations, particularly among individuals without prior depression-related health care experience [43].

The associations between a history of depression diagnosis or treatment and treatment-seeking intention and willingness to take antidepressants should be interpreted with caution. Participants with a previous diagnosis of depression or treatment have already engaged with the health care system; therefore, their greater stated intention to seek treatment and willingness to take antidepressants may partly reflect behavior they have already undertaken rather than an independent finding. Furthermore, given the cross-sectional design, these relationships are open to reverse causation: individuals who were more willing to seek care and accept pharmacotherapy in the first place may have been more likely to visit a physician and receive a diagnosis of depression [44]. Therefore, these findings could be understood as associations describing attitudes observed among individuals who have already engaged in depression care rather than as evidence that prior care increases treatment acceptance.

### Limitations

This study has several limitations. First, participants were recruited using self-selected, nonprobability online convenience sampling, with the survey link openly distributed through social media advertising and messaging app invitations. This strategy is inherently susceptible to selection bias: individuals who are more active online or have a preexisting interest in mental health may be overrepresented, whereas older adults and those less engaged with online platforms may be underrepresented. Because the response rate could not be calculated, the differences between participants and nonparticipants could not be assessed. Although we adjusted for age and sex in the analyses, such adjustments cannot fully account for these differences; therefore, the observed depression screening positivity and help-seeking attitudes should be interpreted as findings within this self-selected online sample rather than as population-level estimates, and they may not be generalizable to the broader Korean adult population. Second, the cross-sectional design precluded causal and temporal inferences. Third, depression screening positivity in this study was identified using a brief screening instrument with a high-sensitivity, modest-specificity threshold chosen to make the likelihood that individuals with possible depression would be missed as low as possible [32]. Therefore, the observed screening positivity should not be interpreted as an estimate of the prevalence of depressive disorders. Because no diagnostic assessment or measure of symptom severity was administered, we could not determine whether help-seeking attitudes or willingness to take antidepressants differed according to a confirmed diagnosis or depression severity. Additional research using a staged approach in which individuals who screen positive for depression undergo additional diagnostic assessments to confirm their diagnosis is needed. Fourth, comorbidities that increase the risk of depression or influence help-seeking were not assessed. Unmeasured comorbidities may confound the observed associations and independently shape treatment attitudes through prior health care engagement. Fifth, because this study was limited to Korean adults, the generalizability of the findings to other ethnic groups remains unclear. Nevertheless, the use of two brief PRIME-MD–derived screening items in a community-based online sample provided insights into help-seeking intentions and willingness to take antidepressants among adults recruited outside clinical settings.

### Conclusions

In this online survey of Korean adults, 65% (684/1053) of the participants had a positive depression screen. Depression screening positivity was differentially associated with the two treatment attitudes: it was not associated with the intention to seek treatment for depression, whereas it was associated with a lower willingness to use antidepressants. A history of depression diagnosis or treatment was associated with greater treatment-seeking intention and a higher willingness to use antidepressants. Comprehensive approaches should be implemented and evaluated to reduce barriers to depression care initiation, improve depression management literacy, and address concerns about pharmacotherapy, thereby increasing help-seeking and early treatment initiation.

## Supplementary material

10.2196/96178 Multimedia Appendix 1 Survey questionnaire and supplementary tables.

## Abbreviations

aOR: adjusted odds ratio
PRIME-MD: Primary Care Evaluation of Mental Disorders
