# Supplementary material for: Depression Screening Positivity, Treatment-Seeking Intention, and Willingness to Take Antidepressants in a Self-Selected Online Sample of Korean Adults: Cross-Sectional Questionnaire Study
Source: JMIR Form Res. 2026 Nov 11;10:e96178. doi: 10.2196/96178 (PMC13754184; doi:10.2196/96178)
Supplement: Supplementary file 1 [file formative-v10-e96178-s001.docx]

**Supplementary Tables**

**Table S1. Survey questionnaire**

**1.** During the past month, have you often been bothered by feeling down, depressed, or hopeless?

□ Yes	□ No

**2.** During the past month, have you often been bothered by little interest or pleasure in doing things?

□ Yes	□ No

**3.** If you were diagnosed with depression, would you be willing to receive treatment at a hospital?

□ Yes	□ No

*Only respondents who answered "No" to item 3 completed item 3-1.*

**3-1.** What are the reasons you would not seek hospital treatment? (Multiple responses allowed)

□ I cannot accept a diagnosis of depression.

□ I believe I can handle it on my own.

□ I am concerned about negative views or stigma from others.

□ I am worried that it could interfere with my school or work life.

□ I am concerned that having a medical record could cause problems later (e.g., when applying for insurance).

□ I do not think that treatment at a hospital would be effective.

□ I do not have a trusted hospital/clinic or regular physician.

□ I am concerned about medication side effects.

□ I lack the time or financial resources for treatment.

**4.** If a doctor told you that you should take antidepressants to treat your depression, would you be willing to take them?

□ Yes	□ No

*Only respondents who answered "No" to item 4 completed item 4-1.*

**4-1.** What are the reasons you would not take antidepressants? (Multiple responses allowed)

□ I am concerned about medication side effects.

□ I am concerned about negative views or stigma from others regarding antidepressant use.

□ I prefer non-pharmacological treatments (e.g., psychotherapy) over antidepressants.

□ I feel reluctant or anxious about taking antidepressants.

□ I am concerned that having a medical record could cause problems later (e.g., when applying for insurance).

□ I am concerned that antidepressants might be addictive or lead to dependence.

**5.** Have you ever been diagnosed with or received treatment for depression in the past?

□ Yes	□ No

**6.** What is your sex?

□ Male	□ Female

**7.** What is your age?

□ 19 years or younger

□ 20–29 years

□ 30–39 years

□ 40–49 years

□ 50–59 years

□ 60 years or older

**Table S2. Within-participant comparison of the associations of depression screening status with treatment-seeking intention and willingness to take antidepressants**

| **Outcome or contrast** | **Effect estimate (95% CI)^1^** | ***P*** |
| --- | --- | --- |
| Treatment-seeking intention for depression: positive vs negative screen | aOR 1.04 (0.76–1.41) | .815 |
| Willingness to take antidepressants: positive vs negative screen | aOR 0.73 (0.54–0.99) | .046 |
| Difference between the two outcome-specific associations: willingness to take antidepressants relative to treatment-seeking intention | ROR 0.70 (0.53–0.94) | .018^2^ |

Values are presented as adjusted odds ratios (aORs) or ratio of ORs (RORs) with 95% confidence intervals (CIs).

^1^aORs and ROR were estimated using a repeated-outcome generalized estimating equations logistic model accounting for within-participant correlation.

^2^*P* value for interaction.

Abbreviations: aOR, adjusted odds ratio; CI, confidence interval; ROR, ratio of odds ratios.

**Table S3. Joint patterns of treatment-seeking intention and willingness to take antidepressants according to depression screening status**

| **Joint response pattern** | **Negative screen, n (%)** | | **Positive screen, n (%)** | |
| --- | --- | --- | --- | --- |
| Treatment-seeking intention: yes / Antidepressant willingness: yes | 260 (70.5) | 454 (66.4) | |  |
| Treatment-seeking intention: yes / Antidepressant willingness: no | 23 (6.2) | 75 (11.0) | |  |
| Treatment-seeking intention: no / Antidepressant willingness: yes | 35 (9.5) | 47 (6.9) | |  |
| Treatment-seeking intention: no / Antidepressant willingness: no | 51 (13.8) | 108 (15.8) | |  |

Values are numbers (column percentages) of participants within each screening group.

**Table S4. Willingness to take antidepressants according to history of depression diagnosis or treatment among participants with a positive depression screen who intended to seek treatment**

| Willingness to take antidepressants | No history of depression diagnosis or treatment (n=376), n (%) | History of depression diagnosis or treatment (n=153), n (%) |
| --- | --- | --- |
| Yes | 313 (83.2) | 141 (92.2) |
| No | 63 (16.8) | 12 (7.8) |

Values are presented as n (% within each column).
